# Pulmonary Symptoms and Psychological Distress as Correlates and Mediators of Quality of Life in Lung Transplant Recipients: A Cross-Sectional Study

**DOI:** 10.3390/jcm15135212

**Published:** 2026-07-03

**Authors:** Aleksandra Stańska, Wojciech Karolak, Sławomir Żegleń, Jacek Wojarski

**Affiliations:** 1 Division of Quality of Life Research, Department of Psychology, Faculty of Health Sciences, Medical University of Gdańsk, 80-210 Gdańsk, Poland; 2 Department of Cardiac & Vascular Surgery, Faculty of Medicine, Medical University of Gdańsk, 80-210 Gdańsk, Poland; 3 Division of Pulmonology, Faculty of Medicine, Medical University of Gdańsk, 80-210 Gdańsk, Poland

**Keywords:** anxiety, depression, lung transplantation, quality of life, patient-reported outcome measures

## Abstract

**Background:** Lung transplant recipients often live for years with residual respiratory symptoms and psychological distress, but the pathways through which these factors affect quality of life (QoL) are not fully understood. We examined how transplant-specific pulmonary symptom burden and psychological distress relate to generic and transplant-specific QoL in long-term lung transplant recipients. **Methods:** In this cross-sectional study, 76 adult lung transplant recipients from a single center completed the Lung Transplant Quality of Life (LT-QoL) questionnaire, EQ-5D-5L, SF-36, St George’s Respiratory Questionnaire (SGRQ), and Hospital Anxiety and Depression Scale (HADS). A composite psychological distress index was derived from HADS-Anxiety, HADS-Depression, and the LT-QoL Anxiety/Depression and Health Distress subscales. Associations were examined using Pearson correlations, hierarchical linear regression (adjusting for age, sex, and time since transplant), and statistical mediation models examining psychological distress as a potential mediator between pulmonary symptoms and QoL outcomes. **Results:** Pulmonary symptom burden (LT-QoL Pulmonary Symptoms) was in the low–moderate range yet showed robust correlations with poorer generic, transplant-specific, and respiratory-specific QoL (|r| up to 0.82). The psychological distress index demonstrated good internal consistency (α = 0.84) and was strongly associated with worse EQ-5D, SF-36, and LT-QoL General QoL scores. In regression models, pulmonary symptoms and psychological distress independently predicted SF-36 overall QoL (*R*^2^ = 0.55), whereas psychological distress was the stronger predictor of the EQ-5D Index Value. Statistical mediation analyses were consistent with partial mediation of the association between pulmonary symptoms and SF-36 and the EQ-5D Index Value, while effects on the EQ-VAS and LT-QoL General QoL were largely direct. **Conclusions:** Even modest pulmonary symptom burden and psychological distress are tightly linked to QoL years after lung transplantation. Routine follow-up should include brief assessment of both domains, and integrated care models that combine optimization of pulmonary status with targeted psychological support may be needed to preserve long-term QoL in lung transplant recipients.

## 1. Background

Lung transplantation is a life-saving treatment for selected patients with end-stage respiratory disease, but long-term outcomes extend well beyond survival [1,2,3,4,5]. Recipients often live for many years with a complex burden of residual symptoms, treatment-related side effects, and psychosocial challenges, all of which can substantially affect health-related quality of life (HRQoL) [1,2,3]. Even in clinically stable patients with satisfactory graft function, limitations in physical capacity, persistent respiratory complaints, and chronic treatment burden may compromise daily functioning and subjective well-being [1,2,3,6]. As a result, international guidelines increasingly emphasize that routine follow-up after lung transplantation should include systematic assessment of patient-reported outcomes (PROs) such as symptoms and QoL, not just traditional clinical or physiological parameters [2,5]. Systematic reviews highlight substantial heterogeneity in concepts, instruments, and the timing of HRQoL assessments after LTx, which complicates synthesis and benchmarking across studies [1,6,7].

Respiratory symptoms remain central to patients’ post-transplant experience. Dyspnea, cough, and activity-related breathing discomfort may persist despite objectively improved lung function [1,2,3,6]. These symptoms can reflect chronic allograft dysfunction, comorbid disease, deconditioning, or treatment-related factors, and they are strongly linked to limitations in physical and social functioning [1,2,3,6]. Disease-specific instruments such as the Lung Transplant Quality of Life (LT-QoL) questionnaire and the St George’s Respiratory Questionnaire (SGRQ) were developed to capture such symptom burden and its impact on daily life in a more granular way than generic QoL measures [8,9,10,11]. However, less is known about how transplant-specific symptom burden relates simultaneously to generic QoL indices, disease-specific respiratory health status, and transplant-specific global QoL within the same cohort [1,2,3,6,9].

Psychological factors are another key component of post-transplant outcomes. Anxiety, depressive symptoms, and health-related worry are common after solid organ transplantation and have been consistently associated with poorer QoL, greater functional impairment, and worse perceived health status [3,8,12,13,14,15,16,17,18]. A recent overview of systematic reviews concluded that although a range of psychological interventions and self-adjustment strategies appear promising for lung transplant recipients, the overall methodological quality of the evidence remains modest [8]. In lung transplant recipients, psychological distress may arise from pre-transplant disease trajectories, prolonged hospitalization, fear of rejection or infection, and chronic treatment demands [3,6,12,13,14,15,16,17]. Screening tools such as the Hospital Anxiety and Depression Scale (HADS) are widely used to quantify anxiety and depressive symptoms in this population [19,20,21]. In addition, transplant-specific instruments like the LT-QoL include domains that capture health-related distress and emotional reactions to life after transplantation [9,22]. Despite this, the extent to which psychological distress helps to explain the impact of ongoing pulmonary symptom burden on QoL after lung transplantation is not fully understood [3,8,12,13,14,15,16,17,18].

Most previous studies have examined either the association between respiratory status and QoL or the association between psychological distress and QoL, often focusing on a single generic measure such as the SF-36 or EQ-5D [1,2,3,4,6,12,20,23,24,25,26]. Fewer analyses have integrated disease-specific symptom burden, transplant-specific QoL, generic QoL, and psychological distress within a single analytic framework. In particular, it remains unclear whether the relationship between pulmonary symptoms and QoL is largely direct or whether it is partly transmitted through heightened psychological distress [3,8,12,13,14,15,16,17,18]. Understanding these pathways is clinically important because it may clarify whether interventions should focus primarily on symptom control, on psychological support, or on both in combination.

The present study addresses this gap by examining how transplant-specific pulmonary symptom burden relates to multiple indicators of QoL in a cohort of adult lung transplant recipients and by testing the mediating role of psychological distress. Using the Polish adaptation of the LT-QoL questionnaire together with the EQ-5D, SF-36, SGRQ, and HADS [9,10,11,19,20,21,23,24,25,27,28], we first describe the levels of symptom burden, psychological distress, and QoL in this sample and their bivariate associations. We then use hierarchical regression models to evaluate the independent contributions of pulmonary symptoms and psychological distress to generic and transplant-specific QoL, controlling for age, sex, and time since transplantation. Finally, we apply mediation models to test whether psychological distress statistically mediates the association between pulmonary symptom burden and different QoL outcomes. These analyses are based on the same clinical cohort that was previously used for the linguistic and psychometric validation of the Polish LT-QoL (preprint, in review; [22]), but the present work addresses distinct mechanistic questions about the interplay between symptoms, psychological distress, and QoL after lung transplantation.

The novelty of the present study lies in the simultaneous use of an expanded set of validated patient-reported outcome measures, including LT-QoL, SGRQ, EQ-5D-5L, SF-36 and HADS, within a single cohort of lung transplant recipients. This approach allows for transplant-specific pulmonary symptoms, generic health status, respiratory-specific health impairment, and psychological distress to be examined within one analytical framework. In contrast to earlier, more fragmented studies focusing on selected instruments or isolated associations, the present study explores both direct associations and statistical indirect pathways linking pulmonary symptom burden, psychological distress, and multiple QoL outcomes. These analyses also place post-transplant quality of life within the broader context of chronic respiratory diseases, such as COPD, in which respiratory limitation and psychological distress are well-recognized determinants of impaired health-related quality of life.

## 2. Methods

### Study Design and Participants

This cross-sectional study was conducted in adult lung transplant recipients followed at the University Clinical Center in Gdańsk, Poland. The present analyses are based on the same clinical cohort that was used for the linguistic and psychometric validation of the Polish version of the LT-QoL questionnaire, reported in detail elsewhere [22]. The validation paper focused on translation procedures, factor structure, and reliability of the LT-QoL. In contrast, the current study addressed distinct, pre-specified research questions about associations between pulmonary symptom burden, psychological distress, and multiple indicators of quality of life. None of the regression or mediation models, hypotheses, or outcome tables presented here were reported in the validation paper, so there is no overlap in the main analytic results.

Eligible participants were adult lung transplant recipients who were attending routine outpatient follow-up during the recruitment period. Recruitment and questionnaire data collection were conducted between December 2022 and July 2023. Consecutive patients seen in the transplant clinic were invited to participate. In addition, patients without a scheduled visit in the near future were contacted by telephone or e-mail and could complete the questionnaires electronically or on paper and return them by post.

A total of 76 lung transplant recipients provided core sociodemographic and clinical data (age, sex, and time since transplantation). Due to missing questionnaire responses, some analyses were conducted on slightly smaller subsamples; exact sample sizes for each instrument and analysis are reported in the tables. For the main analyses linking pulmonary symptoms, psychological distress, and quality of life, 67 participants had complete data for the LT-QoL Pulmonary Symptoms subscale, the psychological measures, and at least one quality-of-life outcome.

Participation was voluntary. Patients provided informed consent prior to completing the questionnaires, either in written form (for paper questionnaires completed during clinic visits) or orally/electronically (for questionnaires completed remotely). The study was exploratory and non-interventional and relied primarily on self-report questionnaire data supplemented with basic anonymized clinical information from medical records. The study was conducted in accordance with the principles of the Declaration of Helsinki.

## 3. Measures

All questionnaires used in this study were previously developed and validated instruments; no study-specific questionnaire was created for this project. The LT-QoL, EQ-5D-5L, SF-36, SGRQ, and HADS were used in their validated Polish versions as referenced below.

### 3.1. Sociodemographic and Clinical Variables

Age, sex, and time since lung transplantation (in months) were extracted from medical records and treated as covariates in all multivariable models.

### 3.2. Lung Transplant Quality of Life Questionnaire (LT-QoL)

Disease-specific quality of life and symptom burden were assessed with the Polish version of the Lung Transplant Quality of Life (LT-QoL) questionnaire, originally developed by Singer and colleagues for lung transplant recipients [9,22]. The LT-QoL covers a broad range of domains relevant after lung transplantation, including symptom burden, functional limitations, emotional concerns, and overall quality of life [9,22]. It comprises first-order subscales assessing specific domains such as Pulmonary Symptoms (Shortness of Breath and Cough), Gastrointestinal Symptoms, Neuromuscular Symptoms, Treatment Burden, Worry About Future Health, Cognitive Limitations, Sexual Problems, Anxiety and Depression, Health Distress, and General Quality of Life [9]. Several second order composite scales (for example, Pulmonary Symptoms, Gastrointestinal Symptoms, and Anxiety/Depression) can be derived by averaging conceptually related subscales.

In the present study, two LT-QoL domains were of primary interest:

**LT-QoL Pulmonary Symptoms**: This is a composite index capturing the severity of respiratory symptoms, calculated as the mean of the Shortness of Breath and Cough subscales. Scores range from 1 to 5, with higher scores indicating more severe pulmonary symptom burden.

**LT-QoL General Quality of Life**: This is a single subscale assessing overall quality of life after lung transplantation. Unlike the symptom-oriented LT-QoL scales, higher scores on this subscale indicate better global quality of life (range 1 to 5).

In addition, two emotion-related LT-QoL subscales—**Anxiety/Depression** and **Health Distress**—were used as transplant-specific indicators of emotional burden and entered, together with HADS-Anxiety and HADS-Depression, into the composite psychological distress index (see Statistical Analyses) [9,19,20,21,22].

All LT-QoL items and subscales were scored according to the original authors’ recommendations [9]. The linguistic adaptation and psychometric validation of the Polish LT-QoL are described in detail elsewhere; the present work uses the same scoring rules to ensure full comparability with prior studies [22].

### 3.3. Hospital Anxiety and Depression Scale (HADS)

Psychological symptoms were assessed with the Hospital Anxiety and Depression Scale (HADS), a widely used 14-item screening instrument designed for use in medical settings [19,20]. The HADS consists of two seven-item subscales measuring anxiety (HADS Anxiety) and depressive symptoms (HADS Depression). Items are rated on 4-point scales from 0 to 3, yielding subscale scores from 0 to 21, with higher scores indicating more severe symptomatology.

The officially adapted Polish version of the HADS was used. Previous Polish validation studies have demonstrated satisfactory internal consistency, factorial validity, and convergent validity in medical and non-medical populations [21]. In the present study, HADS Anxiety and HADS Depression scores were calculated according to standard scoring rules, without any modifications.

### 3.4. Psychological Distress Index

To capture overall psychological distress in a way that integrates symptom-specific and transplant-specific emotional burden, a composite psychological distress index was constructed. Four indicators were used:

HADS Anxiety;

HADS Depression;

LT-QoL Anxiety/Depression subscale;

LT-QoL Health Distress subscale.

First, each of the four subscales was z standardized (mean 0, standard deviation 1). The psychological distress index was then calculated as the mean of these four z scores, with higher values indicating greater psychological distress.

The internal consistency of the four indicators used to derive the psychological distress index was good. In the current sample (*N* = 67 with complete data), Cronbach’s alpha based on standardized subscale scores was 0.84, and inter-correlations among the indicators ranged from *r* = 0.34 to *r* = 0.74, supporting the use of a single composite index.

Conceptually, the composite index was intended to capture a transdiagnostic dimension of emotional burden that cuts across anxiety, depressive symptoms, and transplant-specific health-related worry rather than focusing on any single symptom cluster. Combining generic (HADS) and transplant-specific (LT-QoL) indicators was expected to improve content validity and reduce measurement error associated with any one scale. In a sensitivity analysis, we also examined models with HADS Anxiety and HADS Depression entered as separate mediators. Given the largely similar pattern of findings and the more parsimonious nature of the composite index, we retained the psychological distress index as the primary mediator in the main analyses.

Because the LT-QoL Anxiety/Depression and Health Distress subscales are conceptually related to quality-of-life constructs, analyses involving the composite psychological distress index should be interpreted with appropriate caution. To evaluate the robustness of the findings, additional sensitivity analyses were conducted using HADS Anxiety and HADS Depression separately, without the inclusion of LT-QoL emotional domains. The overall pattern of findings remained similar, supporting the stability of the observed associations.

### 3.5. EQ-5D-5L

Generic health status was assessed using the EQ-5D 5L, developed by the EuroQol Group [25]. The EQ-5D-5L describes health across five dimensions (mobility, self-care, usual activities, pain/discomfort, and anxiety/depression), each rated on five levels of severity. These five responses form a five-digit health state profile. In addition, respondents rate their current overall health on a visual analogue scale (EQ-VAS) from 0 (worst imaginable health) to 100 (best imaginable health) [25].

For the present study, EQ-5D-5L health states were converted into a single index value (EQ-5D Index Value) using the Polish value set based on time trade-off valuations in a representative national sample [27,28]. Higher EQ-5D Index Value and EQ-VAS scores indicate better overall health status. The Polish adaptation and value set studies have shown good measurement properties and support the use of EQ-5D-5L in clinical and population research in Poland.

### 3.6. SF-36 Health Survey

Global health-related quality of life was measured using the Polish version of the 36-Item Short Form Health Survey (SF-36) [23,24]. The SF-36 covers eight domains: physical functioning, role limitations due to physical health, bodily pain, general health perceptions, vitality, social functioning, role limitations due to emotional problems, and mental health [10]. Items are scored and transformed to 0–100 scales, where higher scores typically indicate better health.

Following the Polish scoring approach proposed by Tylka [24], items were first recoded using the Polish SF-36 key so that higher item scores reflect more negative evaluations or more frequent complaints. For each of the eight domains, item scores were summed and linearly transformed to a 0 to 100 scale, with higher domain scores indicating poorer health-related quality of life. To obtain a global index, we calculated the arithmetic mean of the eight domain scores, referred to in this study as the SF-36 overall health-related QoL score, where higher values indicate worse health status. This composite index was used as the primary SF-36 outcome in correlational, regression, and mediation analyses.

### 3.7. St George’s Respiratory Questionnaire (SGRQ)

Disease-specific respiratory health-related quality of life was assessed with the St George’s Respiratory Questionnaire (SGRQ) [10,11]. The SGRQ yields three component scores (Symptoms, Activity, and Impacts) and a Total score, each ranging from 0 to 100, where higher scores denote more severe respiratory health impairment [10].

The officially adapted Polish version of the SGRQ was administered and scored according to the SGRQ manual. In the current study, the SGRQ Total score was used descriptively and in bivariate analyses as a disease-specific comparator for LT-QoL Pulmonary Symptoms; due to the smaller subsample with available SGRQ data, it was not included in multivariable regression or mediation models.

### 3.8. Statistical Analyses

All analyses were conducted using IBM SPSS Statistics for Windows, version 23.0 (IBM Corp., Armonk, NY, USA) and JASP, version 0.95.4 (JASP Team, Amsterdam, The Netherlands). Statistical significance was set at *p* < 0.05 (two-tailed). Analyses were based on available cases. For descriptive statistics and correlations, pairwise non-missing data were used; for regression and mediation models, listwise deletion was applied for the variables in each model. Exact sample sizes are reported in the tables.

First, descriptive statistics were calculated for all variables, including means, standard deviations, and observed ranges for continuous variables and counts and percentages for categorical variables. Internal consistency of the indicators that formed the psychological distress index (HADS Anxiety, HADS Depression, LT-QoL Anxiety/Depression, and LT-QoL Health Distress) was evaluated using Cronbach’s alpha based on standardized scores.

Pearson correlation coefficients were computed to examine bivariate associations between pulmonary symptom burden (LT-QoL Pulmonary Symptoms), HADS Anxiety, HADS Depression, the psychological distress index, generic and transplant-specific quality of life measures (EQ-5D Index Value, EQ-VAS, LT-QoL General Quality of Life, and SF-36 overall score), and SGRQ Total.

To assess the independent contributions of pulmonary symptoms and psychological distress to quality of life, hierarchical linear regression analyses were performed for four outcomes: EQ-5D Index Value, EQ-VAS, LT-QoL General Quality of Life, and the SF-36 overall score. For each outcome, age, sex, and time since transplantation (months) were entered as covariates in block 1. LT-QoL Pulmonary Symptoms (pulmonary symptom burden) was entered in block 2, and the psychological distress index was added in block 3. For each final model, unstandardized regression coefficients (*B*), standard errors, standardized coefficients (*β*), *p* values, and model fit indices (*R*^2^, adjusted *R*^2^, *F*, and *p*) were reported.

To further elucidate the interplay between pulmonary symptoms, psychological distress, and quality of life, mediation models were estimated using structural equation modeling in JASP (version 0.95.4). In these models, pulmonary symptom burden was represented by the LT-QoL Pulmonary Symptoms (predictor), psychological distress by the psychological distress index (mediator), and quality of life by each of the four outcomes (EQ-5D Index Value, EQ-VAS, LT-QoL General Quality of Life, and the SF-36 overall score) in turn. Age, sex, and time since transplantation were included as covariates and were allowed to predict the predictor, the mediator, and each outcome.

Parameters were estimated using maximum likelihood. For each path, we report maximum-likelihood point estimates, standard errors, *z* values, *p* values, and 95% bias-corrected bootstrap confidence intervals based on 5000 resamples. For indirect effects, statistical significance was evaluated primarily based on the bootstrap confidence intervals; indirect effects were considered statistically significant when the 95% confidence interval did not include zero.

As a sensitivity analysis, an additional parallel mediator model was estimated in which the HADS Anxiety and HADS Depression subscale scores were entered as separate mediators between pulmonary symptom burden and quality of life outcomes. This model was used to explore whether anxiety and depressive symptoms contributed differentially to the associations of pulmonary symptom burden with quality of life. Given the largely similar pattern of findings and the potential concern regarding construct overlap, detailed results of the parallel mediator model are summarized narratively as a sensitivity analysis.

Given the exploratory nature of the study and the modest sample size, we did not apply formal corrections for multiple testing. Instead, we interpreted patterns of associations across outcomes and models rather than relying on isolated *p* values close to the 0.05 threshold. The mediation models were estimated in cross-sectional data and therefore should be interpreted as tests of statistical indirect effects rather than evidence of causal mediation. The hypothesized direction from pulmonary symptom burden to psychological distress and quality of life was based on clinical plausibility and the prior literature; however, reverse and bidirectional relationships are also possible. Consequently, the mediation analyses were intended to evaluate whether the observed associations were consistent with theoretically plausible pathways rather than to establish temporal ordering or causal mechanisms.

## 4. Results

### 4.1. Sample Characteristics

The final sample comprised 76 lung transplant recipients (55 men, 72.4%, and 21 women, 27.6%). The mean age was *M* = 57.57 years (*SD* = 12.92, range 25 to 77), and the mean time since lung transplantation was *M* = 56.59 months (*SD* = 30.35, range 33 to 197; Table 1).

On the transplant-specific LT-QoL Pulmonary Symptoms subscale (higher scores indicating more severe respiratory symptoms), the mean score was low to moderate (*M* = 2.05, *SD* = 0.89, range 1.00 to 5.00). The psychological distress index, a standardized composite of HADS-Anxiety, HADS-Depression, LT-QoL Anxiety/Depression, and LT-QoL Health Distress subscales (each z-standardized and then averaged), was centered around zero (*M* = 0.00, *SD* = 0.82, range −1.00 to 2.17). Generic health-related quality of life was relatively preserved: the mean EQ-5D Index Value was *M* = 0.92 (*SD* = 0.12), and the mean EQ-5D VAS was *M* = 75.21 (*SD* = 18.17). Transplant-specific general quality of life (LT-QoL General QoL) was also high (*M* = 4.18, *SD* = 0.92).

Higher scores on the SF-36 overall health-related QoL index and on the SGRQ Total score reflected worse health status; the mean SF-36 overall score was *M* = 54.17 (*SD* = 29.82), and the mean SGRQ Total score was *M* = 10.14 (*SD* = 6.91, *N* = 37). Sample sizes varied across measures due to missing questionnaire data; exact n values for each variable are reported in Table 1.

Primary indications for lung transplantation in this clinical cohort were similar to those reported in our previous LT-QoL validation study from the same center [22]. In that validation sample, the most frequent indications were chronic obstructive pulmonary disease (27 patients, 38.6%) and idiopathic pulmonary fibrosis (15, 21.4%), followed by allergic alveolitis (6, 8.6%), pulmonary arterial hypertension (6, 8.6%), post-COVID-19 respiratory failure (5, 7.1%), sarcoidosis (3, 4.3%), systemic sclerosis (2, 2.9%), rheumatoid arthritis (2, 2.9%), histiocytosis (1, 1.4%), silicosis (1, 1.4%), and other rare interstitial or occupational lung diseases (2, 2.9%). The present analytic sample is nested within this clinical cohort and therefore reflects a comparable diagnostic spectrum.

### 4.2. Bivariate Associations

For clarity, higher scores on the SF-36 overall score indicate worse health-related quality of life, whereas higher scores on EQ-5D outcomes and LT-QoL General Quality of Life indicate better health status and quality of life.

Pearson correlations are presented in Table 2. Higher pulmonary symptom burden was consistently associated with more psychological symptoms and poorer quality of life.

Pulmonary symptom burden correlated positively with HADS-Anxiety (*r* = 0.29, *p* = 0.018) and HADS-Depression (*r* = 0.39, *p* = 0.001), as well as with the psychological distress index (*r* = 0.31, *p* = 0.011). Greater symptom burden was related to worse generic QoL on both EQ-5D measures (EQ-5D Index Value: *r* = −0.38, *p* = 0.001; EQ-5D VAS: *r* = −0.65, *p* < 0.001) and to lower transplant-specific general QoL (*r* = −0.43, *p* < 0.001).

Regarding the generic SF-36 measure, higher pulmonary symptom burden was strongly associated with poorer health-related QoL (*r* = 0.58, *p* < 0.001; higher scores indicate more impairment). In the subsample with SGRQ data, pulmonary symptom burden showed a very strong correlation with SGRQ Total (*r* = 0.82, *p* < 0.001), indicating close agreement between transplant-specific symptom reports and disease-specific respiratory health status.

The psychological distress index showed the expected pattern of correlations: higher distress was associated with a lower EQ-5D Index Value (*r* = −0.57, *p* < 0.001), lower EQ-5D VAS (*r* = −0.37, *p* = 0.002), lower transplant-specific general QoL (*r* = −0.28, *p* = 0.022), and worse SF-36 health-related QoL (*r* = 0.57, *p* < 0.001).

### 4.3. Hierarchical Regression Analyses

To examine independent contributions of pulmonary symptoms and psychological distress, a series of hierarchical linear regression models was conducted with age, sex, and time since transplant entered as covariates in the first block, pulmonary symptom burden (LT-QoL Pulmonary Symptoms) in the second block, and the psychological distress index in the third block. Only the final models are summarized below (Table 3).

### 4.4. EQ-5D Index Value

For the EQ-5D Index Value, the final model explained 39.5 percent of the variance (*R*^2^ = 0.395, adjusted *R*^2^ = 0.346, *F*(5, 61) = 7.98, *p* < 0.001). In the fully adjusted model, higher pulmonary symptom burden showed a trend level association with lower EQ-5D Index Value (*B* = −0.029, *SE* = 0.015, *β* = −0.22, *p* = 0.055), whereas the psychological distress index emerged as a robust independent predictor (*B* = −0.075, *SE* = 0.015, *β* = −0.52, *p* < 0.001). Demographic and clinical covariates were not significant.

### 4.5. EQ-5D VAS

For the EQ-5D VAS, the final model accounted for 49.1 percent of the variance (*R*^2^ = 0.491, adjusted *R*^2^ = 0.449, *F*(5, 61) = 11.77, *p* < 0.001). Greater pulmonary symptom burden was strongly associated with lower self-rated health (*B* = −10.57, *SE* = 2.10, *β* = −0.52, *p* < 0.001). The psychological distress index showed a weaker but borderline significant association (*B* = −4.21, *SE* = 2.13, *β* = −0.19, *p* = 0.053). None of the covariates reached statistical significance in the final model. For illustration, in this model, a one-point increase in LT-QoL Pulmonary Symptoms on its 1 to 5 scale was associated with an approximately 11-point lower rating of current health on the 0-to-100 EQ-VAS, which is likely to be clinically meaningful at the individual patient level.

### 4.6. Transplant-Specific General QoL (LT-QoL General QoL)

For the LT-QoL General QoL scale, the final model explained 25.3 percent of the variance (*R*^2^ = 0.253, adjusted *R*^2^ = 0.191, *F*(5, 61) = 4.13, *p* = 0.003). Higher pulmonary symptom burden was significantly associated with lower transplant-specific general QoL (*B* = −0.379, *SE* = 0.129, *β* = −0.37, *p* = 0.005). The psychological distress index showed a smaller and non-significant association (*B* = −0.213, *SE* = 0.131, *β* = −0.19, *p* = 0.110). Age, sex, and time since transplant were again not significant predictors.

### 4.7. Generic Health-Related QoL (SF-36)

For the SF-36 overall health-related QoL index (higher scores indicating worse status), the final model explained 54.7 percent of the variance (*R*^2^ = 0.547, adjusted *R*^2^ = 0.509, *F*(5, 60) = 14.50, *p* < 0.001). Both pulmonary symptom burden and psychological distress were independently related to worse health-related QoL. Pulmonary symptom burden had a large effect (*B* = 13.39, *SE* = 3.28, *β* = 0.40, *p* < 0.001), and the psychological distress index contributed an additional, similarly strong effect (*B* = 16.44, *SE* = 3.30, *β* = 0.46, *p* < 0.001). Covariates did not reach conventional significance.

## 5. Mediation Analyses

To further clarify the interplay between pulmonary symptoms, psychological distress, and quality of life, path models were estimated within a structural equation modelling framework, with psychological distress tested as a mediator between pulmonary symptom burden and quality-of-life outcomes. In these models, pulmonary symptom burden was entered as a z-standardized LT-QoL Pulmonary Symptoms score, psychological distress was represented by the psychological distress index, and age, sex, and time since transplant were included as covariates. Parameter estimates are summarized in Table 4.

Pulmonary symptom burden was positively associated with psychological distress (path: pulmonary symptom burden → psychological distress index, *B* = 0.259, *SE* = 0.113, *z* = 2.29, *p* = 0.022). In turn, higher psychological distress was strongly related to worse SF-36 health-related QoL (*B* = 16.45, *SE* = 3.13, *z* = 5.26, *p* < 0.001) and a lower EQ-5D Index Value (*B* = −0.075, *SE* = 0.022, *z* = −3.42, *p* < 0.001).

For SF-36, there was a significant indirect effect of pulmonary symptom burden on health-related QoL through psychological distress (*B* = 4.26, *SE* = 1.87, *z* = 2.28, *p* = 0.023, 95 percent CI 0.93 to 9.84), in addition to a substantial direct effect (*B* = 11.94, *SE* = 3.15, *z* = 3.79, *p* < 0.001). The total effect on SF-36 was large (*B* = 16.20, *SE* = 3.14, *z* = 5.16, *p* < 0.001).

For the EQ-5D Index Value, the total effect of pulmonary symptom burden was significant (*B* = −0.045, *SE* = 0.014, *z* = −3.16, *p* = 0.002), and the indirect effect via psychological distress was also significant (*B* = −0.019, *SE* = 0.009, *z* = −2.27, *p* = 0.023), whereas the direct effect in the mediation model did not reach conventional significance (*B* = −0.026, *SE* = 0.015, *p* = 0.077).

For the EQ-5D VAS and the LT-QoL General QoL scale, total effects of pulmonary symptom burden remained significant, whereas indirect effects via psychological distress were smaller and less consistent across inferential criteria (Table 4).

In an additional parallel mediator model, the HADS-Anxiety and HADS-Depression subscales were entered separately as mediators, without inclusion of the LT-QoL emotional domains. The overall pattern of findings was similar to the primary mediation model. The total indirect effect of pulmonary symptom burden remained significant for SF-36 overall QoL (*B* = 4.47, *SE* = 1.92, *p* = 0.020, 95% CI 0.45 to 9.56) and the EQ-5D Index Value (*B* = −0.017, *SE* = 0.008, *p* = 0.037, 95% CI −0.045 to −0.001) but not for the EQ-VAS or LT-QoL General QoL. Anxiety appeared to carry most of the indirect association, whereas the indirect paths through depression did not reach statistical significance.

Across all models, age, sex, and time since transplant were included as covariates and did not show consistent independent associations with quality-of-life outcomes.

## 6. Discussion

This study examined how transplant-specific pulmonary symptom burden and psychological distress are linked to multiple indicators of quality of life in long-term lung transplant recipients. Three main findings emerged. First, higher pulmonary symptom burden was consistently associated with poorer quality of life across generic, transplant-specific, and respiratory-specific measures. Second, a composite psychological distress index showed strong associations with quality of life, particularly with generic health-related quality of life. Third, statistical mediation analyses suggested that psychological distress may partly account for the association between pulmonary symptom burden and selected quality-of-life outcomes, especially the EQ-5D Index Value and the SF-36 overall score.

### 6.1. Pulmonary Symptom Burden and Quality of Life

The observed levels of pulmonary symptoms were in the low to moderate range on average, yet even within this restricted spectrum, symptom burden was robustly related to quality of life. Higher scores on the LT-QoL Pulmonary Symptoms scale were associated with worse EQ-5D outcomes, lower LT-QoL General Quality of Life, poorer SF-36 health status, and markedly worse disease-specific respiratory status on the SGRQ. This pattern is consistent with prior work using the LT-QoL and other instruments, which has shown that residual dyspnea, cough, and respiratory limitations remain key determinants of health-related quality of life after lung transplantation, even among clinically stable survivors [1,2,3,4,6,9,23,24,25,26].

The very strong correlation between LT-QoL Pulmonary Symptoms and SGRQ Total supports the convergent validity of the LT-QoL pulmonary domain as a concise marker of respiratory health impairment [9,10,11,26]. At the same time, the substantial associations with both EQ-5D indices and the SF-36 overall score underline that pulmonary symptoms are not confined to the respiratory domain but permeate patients’ broader perceptions of physical functioning, energy, and everyday life [1,2,3,4,8,9,10,23,24,25,26]. These findings are compatible with systematic reviews showing that although lung transplantation improves health-related quality of life compared with pre-transplant status, many recipients continue to experience persistent limitations several years after surgery, with lung-related symptoms among the strongest correlates of impaired quality of life [1,2,3,6,7].

Importantly, this pattern is not unique to lung transplantation. Across a range of chronic respiratory conditions, including chronic obstructive pulmonary disease and other causes of respiratory failure, symptom burden and psychological distress consistently emerge as major determinants of health-related quality of life. Similar findings were recently reported in patients undergoing video-assisted thoracoscopic plication for unilateral diaphragmatic relaxation, where improvements in respiratory function were accompanied by clinically meaningful gains in SF-36, EQ-5D-5L, and SGRQ outcomes [29]. Together, these observations support the broader concept that respiratory symptoms, emotional well-being and quality of life are closely interconnected regardless of the underlying respiratory diagnosis.

### 6.2. Psychological Distress as a Parallel and Partial Mediator

The psychological distress index, integrating HADS Anxiety and Depression with the LT-QoL Anxiety/Depression and Health Distress subscales, showed good internal consistency (Cronbach’s *alpha* = 0.84 for standardized items) and behaved as a coherent marker of transdiagnostic emotional burden. As expected, higher distress was associated with lower EQ-5D Index Value and EQ-VAS scores, lower LT-QoL General Quality of Life, and worse SF-36 health-related quality of life. This aligns with prior work in lung and other solid organ transplant recipients, where elevated anxiety and depressive symptoms have been repeatedly linked to poorer quality of life and worse self-rated health [3,12,13,14,15,16,17,18].

Earlier studies have also documented that psychosocial vulnerability and distress are associated with more severe physical symptoms and physical impairment [13], more distressing treatment-related symptom experiences and lower adherence [14,17,30], higher rates of nonadherence across organ types [30], and, in some cohorts, higher post-transplant mortality or worse composite outcomes [15,16,18]. A meta-analysis found no robust association between pre-transplant anxiety/depression scores and post-transplant survival [18], which underlines that psychological distress may be more closely tied to functional outcomes and perceived health status than to hard survival endpoints. Our findings add to this literature by explicitly modeling psychological distress as a pathway linking pulmonary symptoms with quality of life rather than treating distress only as a parallel correlate.

In the hierarchical regression models, pulmonary symptom burden and psychological distress made partly independent contributions to quality of life. For the SF-36 overall score, both predictors had large and comparable standardized effects, together explaining over half of the variance. For the EQ-5D Index Value, psychological distress emerged as the more robust independent predictor, whereas for the EQ-VAS and transplant-specific general quality of life, the association with pulmonary symptoms was stronger, and the additional contribution of psychological distress was weaker or borderline significant. This pattern suggests that patients’ cognitively integrated evaluations of health status, such as those captured by the EQ-5D Index Value and SF-36, may be particularly sensitive to emotional distress, whereas more immediate, global self-ratings and disease-specific evaluations may be driven more directly by symptom burden.

The mediation analyses further clarified these relationships. For the SF-36 overall score, psychological distress carried a significant part of the association between pulmonary symptom burden and health-related quality of life, while a substantial direct effect of pulmonary symptoms remained. This is consistent with a dual pathway model, in which respiratory symptoms may be associated with poorer quality of life both directly, through physical limitations and discomfort, and indirectly, by increasing emotional distress that, in turn, colors patients’ perceptions of their health. For the EQ-5D Index Value, the total effect of pulmonary symptom burden was partly explained by the indirect path through psychological distress, and the direct effect in the mediation model fell below conventional significance, consistent with partial mediation. In contrast, for the EQ-VAS and LT-QoL General Quality of Life scales, indirect effects via psychological distress were smaller and did not consistently reach significance, indicating that these outcomes are more tightly linked to symptom burden itself than to distress.

The sensitivity analysis with HADS-Anxiety and HADS-Depression entered as parallel mediators suggests that anxiety may be particularly important in carrying the impact of pulmonary symptoms, whereas depression alone contributes less uniquely when shared variance is accounted for. This is clinically plausible, given that breathlessness and fluctuating respiratory status are prototypical triggers of health-related anxiety and hypervigilance in chronic lung disease [25,31].

At the same time, the present findings should not be interpreted as evidence that psychological distress causally mediates the effects of pulmonary symptoms. Because all variables were assessed at a single timepoint, alternative explanations remain plausible. For example, psychological distress may increase symptom vigilance and symptom reporting, or poorer perceived health status may contribute to elevated distress. The observed indirect effects therefore represent statistical patterns consistent with a potential pathway linking pulmonary symptoms, distress, and quality of life rather than proof of temporal or causal relationships.

An important conceptual consideration is the partial overlap between the psychological distress indicators and some of the quality-of-life outcomes. Both the EQ-5D Index Value and the SF-36 overall score include emotional components, and the LT-QoL Health Distress and Anxiety/Depression subscales capture related content. This conceptual proximity is likely to contribute to the strength of the associations between distress and generic QoL indices and may partly inflate estimates of the indirect effects. At the same time, the persistence of robust associations between pulmonary symptom burden and QoL after adjustment for distress and the similar pattern of findings for more physically oriented outcomes such as the EQ-VAS suggest that the interpretation of distress as a relevant parallel and partial mediator remains clinically meaningful.

These findings also resonate with broader work on psychosocial risk and evaluation in transplantation, including structured tools such as the Stanford Integrated Psychosocial Assessment for Transplantation (SIPAT) [32] and descriptive studies of psychosocial profiles among lung transplant candidates from the same center [26]. Together, these data underscore that psychosocial factors are integral to understanding post-transplant trajectories rather than an optional add-on [3,12,13,14,15,16,17,18,32]. Cluster-analytic work further suggests that a substantial subgroup of lung transplant recipients follows a trajectory of persistently high distress and impaired HRQoL, with little spontaneous improvement over time [1].

### 6.3. Clinical Implications

From a clinical perspective, the findings argue against a purely biomedical focus on lung function and rejection surveillance in long term follow-up. Even among relatively stable survivors several years after transplantation, modest elevations in pulmonary symptom burden and psychological distress were associated with meaningful decrements in quality of life. Systematic assessment of both domains is therefore warranted in routine care, using brief tools such as the LT-QoL pulmonary and emotional subscales together with generic quality-of-life measures (EQ-5D and SF-36) and brief anxiety/depression screens such as the HADS [9,10,11,19,20,21,22,23,24,25].

The partial mediation by psychological distress suggests that interventions targeting distress could attenuate some of the quality-of-life impact of pulmonary symptoms, even if residual symptoms cannot be fully eliminated. Integrated models of care that combine optimization of medical management with psychological support, such as cognitive behavioral strategies for health anxiety, coping with breathlessness, and adjustment to chronic graft-related limitations, may therefore be particularly beneficial. At the same time, the strong direct effects of pulmonary symptoms across outcomes highlight that optimizing pulmonary status, rehabilitation, and symptom management remains fundamental for preserving quality of life [1,2,3,4,6,8,9,10,12,23,24,25,26,33,34].

Emerging concepts of prehabilitation and ongoing rehabilitation in solid organ transplant candidates and recipients, which emphasize a combination of physical training, nutritional optimization, and psychosocial interventions to enhance resilience before and after surgery, are consistent with this dual focus [33,34]. Narrative reviews and consensus statements suggest that such multimodal programs are feasible and may improve functional capacity and HRQoL, although high-quality randomized trials remain limited [33,34].

### 6.4. Strengths and Limitations

Key strengths of this study include the use of a disease-specific instrument tailored to lung transplant recipients, alongside multiple generic and respiratory-specific quality of life measures [1,2,3,4,6,9,10,11,22,23,24,25,26], and the explicit modeling of psychological distress as a composite mediator. Using a composite index allowed us to capture shared variance across anxiety, depression, and health-related distress without relying on arbitrary cut-offs for any single scale [3,8,12,13,14,15,16,17,18,19,20,21,22]. The analytic strategy combined hierarchical regression with structural equation modeling, providing converging evidence on direct and indirect pathways.

Several limitations should be acknowledged. First, the cross-sectional design precludes causal inference. Although the hypothesized direction from pulmonary symptoms to distress to quality of life is theoretically and clinically plausible, reverse and bidirectional influences are also likely [1,2,3,4,6,12,13,14,15,16,17,18,22]. For example, poorer perceived health and impaired HRQoL may increase psychological distress, which, in turn, may heighten symptom perception and symptom reporting. The mediation analyses therefore identify statistical indirect effects that are consistent with a dual pathway model, but they cannot establish temporal ordering or causal mediation.

Second, there is conceptual overlap between the psychological distress index and some of the quality-of-life indices, particularly the EQ-5D Index and the SF-36 overall score, which include emotional components [19,20,21,23,24,25]. This overlap may inflate the strength of associations between distress and generic QoL. However, the robust associations observed between pulmonary symptoms and QoL after adjustment for distress, as well as the similar pattern of results for outcomes that are more strongly driven by physical status such as the EQ-VAS, argue against a purely artefactual explanation [1,2,3,4,6,9,10,11,12,13,14,15,16,17,22,23,24,25,26].

Furthermore, the sample size was modest relative to the complexity of the multivariable models. Although the number of predictors was intentionally limited and bootstrap confidence intervals were used to improve the robustness of parameter estimates, some regression coefficients and indirect effects may still be unstable. Accordingly, the findings should be viewed as exploratory and hypothesis generating until replicated in larger multi-center cohorts.

Third, the sample was recruited from a single transplant center and consisted of relatively long-term survivors, with a mean of nearly five years since transplantation. Patients with early post-transplant complications, those lost to follow-up, and individuals with the most severe impairments are likely under-represented. The recruitment strategy, which relied on attendees at routine outpatient visits and additional invitations by telephone or email, may also have preferentially included more engaged and better functioning patients [1,2,3,4,6,8,22,26]. As a result, the range of symptom burden and distress may be restricted, which would tend to underestimate true associations.

In addition, information on disease duration before transplantation, waiting time between transplant qualification and transplantation, and detailed socioeconomic variables such as household income, living conditions, and family status was not systematically available in the present dataset. These factors may influence long-term adaptation and quality of life after transplantation and should be considered in future prospective studies.

Fourth, key variables were based on self-report questionnaires. Although these instruments have documented reliability and validity [9,10,11,19,20,21,23,24,25], responses may be influenced by current mood and reporting styles. The study did not include concurrent objective indicators such as lung function parameters, six-minute walk distance, chronic lung allograft dysfunction status, or detailed comorbidity profiles, which would allow for more fine-grained modeling of the links between physiological impairment, symptoms, distress, and quality of life [1,2,3,4,6,8,12,15,16,17,26]. Future work should integrate patient reported outcomes with clinical and functional data to disentangle perceived from physiological impairment.

Fifth, the psychological distress index aggregates heterogeneous emotional constructs. This approach improves reliability and parsimony but may obscure potentially important differences between anxiety, depressive symptoms, and health-related worry [3,8,12,13,14,15,16,17,18]. The parallel mediator analysis suggests that anxiety may be more closely tied to the impact of pulmonary symptoms than depression, but these findings require replication in larger samples.

Sixth, the sample size for the structural equation models was modest relative to model complexity. Although we used bootstrap confidence intervals to increase robustness, estimates of indirect effects may still be unstable, and the mediation models should be viewed as exploratory. Finally, we examined multiple outcomes and conducted several related regression and mediation analyses without formal correction for multiple testing. In view of the exploratory nature of the study, we focused on the overall pattern of findings across outcomes and emphasized more robust and consistent effects, but some statistically significant results, particularly those with *p* values close to 0.05, should be interpreted with caution.

### 6.5. Future Directions

Future research should build on these findings in several ways. Longitudinal studies with repeated assessments of symptoms, distress, and quality of life could clarify the temporal dynamics and test whether changes in distress mediate the impact of evolving pulmonary status on subsequent quality of life [1,2,3,4,6,12,15,16,17,18,22]. Incorporating objective clinical indicators and biomarkers would help disentangle perceived from physiological impairment and might identify subgroups in whom subjective distress is disproportionately high relative to clinical status [12,15,16,17,18,20]. Recent studies have also highlighted the potential value of structural and functional assessment of respiratory muscles, including CT-based measures of diaphragm thickness combined with functional testing, as complementary markers of respiratory impairment [35]. Integrating such objective physiological indicators with patient-reported outcomes may improve risk stratification and provide a more comprehensive understanding of the mechanisms linking respiratory dysfunction, psychological distress, and quality of life.

Intervention studies are also needed to examine whether targeted psychological or integrated rehabilitation interventions can effectively reduce distress and improve quality of life among lung transplant recipients with elevated symptom burden, building on existing evidence for pulmonary rehabilitation and cognitive behavioral approaches in chronic lung disease and transplantation [3,12,13,14,15,16,17,18,31,32,33,34]. Finally, multi-center studies using harmonized lung-transplant-specific and generic instruments, including the LT-QoL, EQ-5D, and SF-36, could test the robustness of the present findings across healthcare systems and patient populations and inform the development of routine, low-burden tools for monitoring both physical and psychological outcomes in long-term post-transplant care [1,2,3,4,6,9,22,23,24,25].

### 6.6. Conclusions

Even modest pulmonary symptom burden and psychological distress were closely associated with quality of life in long-term lung transplant recipients. The findings suggest that routine post-transplant follow-up should include brief assessment of both respiratory symptom burden and psychological distress. Although the cross-sectional design precludes causal interpretation, the observed pattern supports integrated care models combining optimization of pulmonary status with targeted psychological support to preserve long-term quality of life after lung transplantation.

## Figures and Tables

**Table 1:** Sample characteristics and descriptive statistics.

Variable		*N*	Minimum	Maximum	*M*	*SD*
Age (years)		76	25.00	77.00	57.57	12.92
Time since lung transplantation (months)		76	33.00	197.00	56.59	30.35
LT-QoL Pulmonary Symptoms (1 to 5; higher = more symptoms)		67	1.00	5.00	2.05	0.89
Psychological distress index ^1^		67	−1.00	2.17	0.00	0.82
EQ-5D Index Value (0 to 1; higher = better)		67	0.38	1.00	0.92	0.12
EQ-5D VAS (0 to 100; higher = better)		67	15.00	100.00	75.21	18.17
LT-QoL General Quality of Life (1 to 5; higher = better)		67	1.00	5.00	4.18	0.92
SF-36 overall health-related QoL ^2^		66	7.00	125.00	54.17	29.82
SGRQ Total (0 to 100; higher = worse)		37	0.52	29.57	10.14	6.91
**Categorical variable**	**Category**	***N* (%)**				
Sex	Male	55 (72.4)				
	Female	21 (27.6)				

**Notes.** ^1^ Psychological distress index is a standardized composite score derived from HADS-Anxiety, HADS-Depression, LT-QoL Anxiety/Depression, and LT-QoL Health Distress (each subscale was z-standardized and then averaged; higher scores indicate greater distress). ^2^ Higher SF-36 overall scores reflect worse health-related quality of life.

**Table 2:** Pearson correlations between pulmonary symptoms, psychological distress, and quality-of-life measures.

Variable	1	2	3	4	5	6	7	8	9
1. LT-QoL Pulmonary Symptoms	—	0.29 *	0.39 **	0.31 *	−0.38 **	−0.65 ***	−0.43 ***	0.58 ***	0.82 ***
2. HADS-Anxiety	0.29 *	—	0.62 ***	0.87 ***	−0.50 ***	−0.39 **	−0.23 †	0.54 ***	0.27
3. HADS-Depression	0.39 **	0.62 ***	—	0.77 ***	−0.39 **	−0.38 **	−0.31 *	0.51 ***	0.28 †
4. Psychological distress index ^1^	0.31 *	0.87 ***	0.77 ***	—	−0.57 ***	−0.37 **	−0.28 *	0.57 ***	0.23
5. EQ-5D Index Value	−0.38 **	−0.50 ***	−0.39 **	−0.57 ***	—	0.58 ***	0.28 *	−0.64 ***	−0.52 **
6. EQ-5D VAS	−0.65 ***	−0.39 **	−0.38 **	−0.37 **	0.58 ***	—	0.31 *	−0.66 ***	−0.76 ***
7. LT-QoL General QoL	−0.43 ***	−0.23 †	−0.31 *	−0.28 *	0.28 *	0.31 *	—	−0.54 ***	−0.43 **
8. SF-36 overall QoL ^2^	0.58 ***	0.54 ***	0.51 ***	0.57 ***	−0.64 ***	−0.66 ***	−0.54 ***	—	0.67 ***
9. SGRQ Total	0.82 ***	0.27	0.28 †	0.23	−0.52 **	−0.76 ***	−0.43 **	0.67 ***	—

† *p* < 0.10; *p < 0.05; ** *p* < 0.01; *** *p* < 0.001. **Notes.** ^1^ Psychological distress index is a standardized composite score derived from the HADS-Anxiety, HADS-Depression, LT-QoL Anxiety/Depression, and LT-QoL Health Distress subscales (each subscale was z standardized and then averaged; higher scores indicate greater distress). ^2^ Values are Pearson correlation coefficients. Higher scores on LT-QoL Pulmonary Symptoms and SGRQ Total indicate worse respiratory status; higher scores on the EQ-5D and LT-QoL General QoL reflect better quality of life; and higher SF-36 overall scores indicate more impairment.

**Table 3:** Final hierarchical regression models predicting quality-of-life outcomes.

(a) EQ-5D Index Value				
Predictor	*B*	*SE B*	*β*	*p*
Age (years)	−0.001	0.001	−0.13	0.205
Sex (2 = female)	−0.015	0.028	−0.06	0.595
Time since transplant (months)	−0.0002	0.0004	−0.04	0.688
LT-QoL Pulmonary Symptoms	−0.029	0.015	−0.22	0.055
Psychological distress index	−0.075	0.015	−0.52	<0.001
Model statistics: *R*^2^ = 0.395, adjusted *R*^2^ = 0.346, *F*(5, 61) = 7.98, *p* < 0.001.				
**(b) EQ-5D VAS**				
**Predictor**	***B***	***SE B***	***β***	***p***
Age (years)	0.008	0.141	0.01	0.957
Sex (2 = female)	6.60	3.96	0.16	0.101
Time since transplant (months)	−0.071	0.063	−0.11	0.265
LT-QoL Pulmonary Symptoms	−10.57	2.10	−0.52	<0.001
Psychological distress index	−4.21	2.13	−0.19	0.053
Model statistics: *R*^2^ = 0.491, adjusted *R*^2^ = 0.449, *F*(5, 61) = 11.77, *p* < 0.001.				
**(c) LT-QoL General Quality of Life**				
**Predictor**	***B***	***SE B***	***β***	***p***
Age (years)	−0.015	0.009	−0.20	0.088
Sex (2 = female)	−0.127	0.244	−0.06	0.604
Time since transplant (months)	−0.001	0.004	−0.03	0.773
LT-QoL Pulmonary Symptoms	−0.379	0.129	−0.37	0.005
Psychological distress index	−0.213	0.131	−0.19	0.110
Model statistics: *R*^2^ = 0.253, adjusted *R*^2^ = 0.191, *F*(5, 61) = 4.13, *p* = 0.003.				
**(d) SF-36 overall health related QoL (higher = worse)**				
**Predictor**	***B***	***SE B***	***β***	***p***
Age (years)	0.403	0.220	0.17	0.072
Sex (2 = female)	−5.48	6.16	−0.08	0.377
Time since transplant (months)	0.053	0.097	0.05	0.585
LT-QoL Pulmonary Symptoms	13.39	3.28	0.40	<0.001
Psychological distress index	16.44	3.30	0.46	<0.001
Model statistics: *R*^2^ = 0.547, adjusted *R*^2^ = 0.509, *F*(5, 60) = 14.50, *p* < 0.001.				

**Table 4:** Mediation of the association between pulmonary symptom burden and quality of life by psychological distress. **Predictor:** pulmonary symptom burden (standardized LT-QoL Pulmonary Symptoms score). Mediator: psychological distress index. **Covariates:** age, sex, and time since transplant.

Outcome	Effect Type	*b*	*SE*	*z*	*p*	95% CI Lower	95% CI Upper
**SF-36 overall quality of life**	Direct effect (controlling for psychological distress)	11.94	3.15	3.79	<0.001	4.64	17.08
	Indirect effect via psychological distress	4.26	1.87	2.28	0.023	0.93	9.84
	Total effect	16.20	3.14	5.16	<0.001	9.36	21.77
**EQ-5D Index Value**	Direct effect	−0.03	0.02	−1.77	0.077	−0.05	0.01
	Indirect effect via psychological distress	−0.02	0.01	−2.27	0.023	−0.05	−0.00
	Total effect	−0.05	0.01	−3.16	0.002	−0.09	−0.01
**EQ-5D VAS**	Direct effect	−9.41	2.46	−3.83	<0.001	−13.39	−3.58
	Indirect effect via psychological distress	−1.09	0.68	−1.60	0.109	−3.54	−0.05
	Total effect	−10.50	1.76	−5.97	<0.001	−14.13	−5.58
**LT-QOL General Quality of Life**	Direct effect	−0.34	0.16	−2.12	0.034	−0.65	−0.03
	Indirect effect via psychological distress	−0.06	0.04	−1.41	0.158	−0.21	0.01
	Total effect	−0.39	0.11	−3.66	<0.001	−0.65	−0.12

**Notes.** Pulmonary symptom burden (standardized) was obtained by z-standardizing the LT-QoL Pulmonary Symptoms subscale. The psychological distress index is a standardized composite score derived from HADS-Anxiety, HADS-Depression, LT-QoL Anxiety/Depression, and LT-QoL Health Distress (each subscale z-standardized and averaged; higher scores indicate greater distress). All models adjust for age, sex, and time since transplant. Point estimates and *z* values are based on maximum likelihood estimation, whereas 95 percent confidence intervals are bias-corrected bootstrap intervals as reported by JASP.

## Data Availability

The datasets generated and analyzed during the current study are not publicly available due to patient privacy and institutional data protection regulations but are available from the corresponding author on reasonable request.

## Abbreviations

EQ-5D-5L	EuroQol 5-Dimension, 5-Level questionnaire
EQ-5D Index Value	Preference-based index derived from EQ-5D-5L health states
EQ-VAS	EQ-5D Visual Analogue Scale
HADS	Hospital Anxiety and Depression Scale
HRQoL	Health-related quality of life
LT-QoL	Lung Transplant Quality of Life questionnaire
PROs	Patient-reported outcomes
QoL	Quality of life
SF-36	36-Item Short Form Health Survey
SGRQ	St George’s Respiratory Questionnaire
SIPAT	Stanford Integrated Psychosocial Assessment for Transplantation

## References

[1] Seiler A., Klaghofer R., Ture M., Komossa K., Martin-Soelch C., Jenewein J. (2016). A systematic review of health-related quality of life and psychological outcomes after lung transplantation. J. Heart Lung Transplant..

[2] Singer J.P., Singer L.G. (2013). Quality of life in lung transplantation. Semin. Respir. Crit. Care Med..

[3] Singer J.P., Katz P.P., Soong A., Shrestha P., Huang D., Ho J., Mindo M., Greenland J.R., Hays S.R., Golden J. (2017). Effect of Lung Transplantation on Health-Related Quality of Life in the Era of the Lung Allocation Score: A U.S. Prospective Cohort Study. Am. J. Transplant..

[4] Singh T.P., Hsich E., Cherikh W.S., Perch M., Hayes D., Lewis A., Dellgren G., Cogswell R. (2025). The International Thoracic Organ Transplant Registry of the International Society for Heart and Lung Transplantation: 2025 Annual Report of Heart and Lung Transplantation. J. Heart Lung Transplant..

[5] Weill D., Benden C., Corris P.A., Dark J.H., Davis R.D., Keshavjee S., Lederer D.J., Mulligan M.J., Patterson G.A., Singer L.G. (2015). A consensus document for the selection of lung transplant candidates: 2014—An update from the Pulmonary Transplantation Council of the International Society for Heart and Lung Transplantation. J. Heart Lung Transplant..

[6] Raguragavan A., Jayabalan D., Saxena A. (2023). Health-related Quality of Life Outcomes Following Single or Bilateral Lung Transplantation: A Systematic Review. Transplantation.

[7] Raguragavan A., Jayabalan D., Saxena A. (2023). Health-related quality of life following lung transplantation for cystic fibrosis: A systematic review. Clinics.

[8] Guo S., Jia Y., Wang R., Sun J., Liu H. (2025). Psychological distress and self-psychological adjustment methods of lung transplant recipients: An overview of systematic reviews. BMC Psychol..

[9] Singer J.P., Soong A., Chen J., Shrestha P., Zhuo H., Gao Y., Greenland J.R., Hays S.R., Kukreja J., Golden J. (2019). Development and Preliminary Validation of the Lung Transplant Quality of Life (LT-QOL) Survey. Am. J. Respir. Crit. Care Med..

[10] Jones P.W., Quirk F.H., Baveystock C.M., Littlejohns P. (1992). A self-complete measure of health status for chronic airflow limitation. The St. George’s Respiratory Questionnaire. Am. Rev. Respir. Dis..

[11] Kuźniar T., Patkowski J., Liebhart J., Wytrychowski K., Dobek R., Ślusarz R., Liebhart E., Małolepszy J. (1999). Validation of the Polish version of St. George’s respiratory questionnaire in patients with bronchial asthma. Pneumonol. Alergol. Pol..

[12] Dew M.A., DiMartini A.F., DeVito Dabbs A.J., Fox K.R., Myaskovsky L., Posluszny D.M., Switzer G.E., Zomak R.A., Kormos R.L., Toyoda Y. (2012). Onset and risk factors for anxiety and depression during the first 2 years after lung transplantation. Gen. Hosp. Psychiatry.

[13] De Vito Dabbs A., Dew M.A., Stilley C.S., Manzetti J., Zullo T., McCurry K.R., Kormos R.L., Iacono A. (2003). Psychosocial vulnerability, physical symptoms and physical impairment after lung and heart-lung transplantation. J. Heart Lung Transplant..

[14] Kugler C., Fischer S., Gottlieb J., Tegtbur U., Welte T., Goerler H., Simon A., Haverich A., Strueber M. (2007). Symptom experience after lung transplantation: Impact on quality of life and adherence. Clin. Transplant..

[15] Smith P.J., Blumenthal J.A., Trulock E.P., Freedland K.E., Carney R.M., Davis R.D., Hoffman B.M., Palmer S.M. (2016). Psychosocial Predictors of Mortality Following Lung Transplantation. Am. J. Transplant..

[16] Smith P.J., Snyder L.D., Palmer S.M., Hoffman B.M., Stonerock G.L., Ingle K.K., Saulino C.K., Blumenthal J.A. (2018). Depression, social support, and clinical outcomes following lung transplantation: A single-center cohort study. Transpl. Int..

[17] Wessels-Bakker M.J., van de Graaf E.A., Kwakkel-van Erp J.M., Heijerman H.G., Cahn W., Schappin R. (2022). The relation between psychological distress and medication adherence in lung transplant candidates and recipients: A cross-sectional study. J. Clin. Nurs..

[18] Courtwright A.M., Salomon S., Lehmann L.S., Wolfe D.J., Goldberg H.J. (2016). The Effect of Pretransplant Depression and Anxiety on Survival Following Lung Transplant: A Meta-analysis. Psychosomatics.

[19] Zigmond A.S., Snaith R.P. (1983). The hospital anxiety and depression scale. Acta Psychiatr. Scand..

[20] Snaith R.P. (2003). The Hospital Anxiety And Depression Scale. Health Qual. Life Outcomes.

[21] Mihalca A.M., Pilecka W. (2015). The factorial structure and validity of the Hospital Anxiety and Depression Scale (HADS) in Polish adolescents. Psychiatr. Pol..

[22] Stańska A., Karolak W., Sławomir Z., Singer J.P., Jacek W. (2024). Polish adaptation of the Lung Transplant Quality of Life (LT-QoL) questionnaire. Preprint.

[23] Ware J.E., Sherbourne C.D. (1992). The MOS 36-item short-form health survey (SF-36). I. Conceptual framework and item selection. Med. Care.

[24] Tylka J., Piotrowicz R. (2009). Kwestionariusz oceny jakości życia SF-36—Wersja polska [Quality of life questionnaire SF-36—Polish version]. Kardiol. Pol..

[25] Herdman M., Gudex C., Lloyd A., Janssen M., Kind P., Parkin D., Bonsel G., Badia X. (2011). Development and preliminary testing of the new five-level version of EQ-5D (EQ-5D-5L). Qual. Life Res..

[26] Karolak W., Stańska A., Wojarski J., Shinde R., Ciak E., Polishchuk A., Łojko M., Sheikhsagha E., Ulstrup I.H., Łacka M. (2022). Demographic and Psychosocial Characteristics of Lung Transplant Candidates: Single-Center Analysis. Transpl. Proc..

[27] Golicki D., Jakubczyk M., Graczyk K., Niewada M. (2019). Valuation of EQ-5D-5L Health States in Poland: The First EQ-VT-Based Study in Central and Eastern Europe. Pharmacoeconomics.

[28] Golicki D. (2021). General population reference values for the EQ-5D-5L index in Poland: Estimations using a Polish directly measured value set. Pol. Arch. Intern. Med..

[29] Topolnitskiy E.B., Shefer N.A., Yunusov A.N. (2024). Quality of life in patients with postoperative unilateral diaphragm relaxation. Khirurgiia.

[30] Dew M.A., DiMartini A.F., De Vito Dabbs A., Myaskovsky L., Steel J., Unruh M., Switzer G.E., Zomak R., Kormos R.L., Greenhouse J.B. (2007). Rates and risk factors for nonadherence to the medical regimen after adult solid organ transplantation. Transplantation.

[31] Yohannes A.M., Junkes-Cunha M., Smith J., Vestbo J. (2017). Management of Dyspnea and Anxiety in Chronic Obstructive Pulmonary Disease: A Critical Review. J. Am. Med. Dir. Assoc..

[32] Maldonado J.R., Dubois H.C., David E.E., Sher Y., Lolak S., Dyal J., Witten D. (2012). The Stanford Integrated Psychosocial Assessment for Transplantation (SIPAT): A new tool for the psychosocial evaluation of pre-transplant candidates. Psychosomatics.

[33] Quint E.E., Ferreira M., van Munster B.C., Nieuwenhuijs-Moeke G., Te Velde-Keyzer C., Bakker S.J.L., Annema C., Mathur S., Pol R.A. (2023). Prehabilitation in Adult Solid Organ Transplant Candidates. Curr. Transplant. Rep..

[34] Annema C., De Smet S., Castle E.M., Overloop Y., Klaase J.M., Janaudis-Ferreira T., Mathur S., Kouidi E., Saez M.J.P., Matthys C. (2023). European Society of Organ Transplantation (ESOT) Consensus Statement on Prehabilitation for Solid Organ Transplantation Candidates. Transpl. Int..

[35] Topolnitskiy E.B., Gusakov V.V., Volinsky A.A. (2026). Computed tomography assessment of diaphragm thickness in myasthenia gravis with clinical and functional correlations. Cureus.

